# Colloidal stability and catalytic activity of cerium oxide nanoparticles in cell culture media†

**DOI:** 10.1039/d0ra08063b

**Published:** 2020-10-27

**Authors:** Xiaohui Ju, Anna Fučíková, Břetislav Šmíd, Jaroslava Nováková, Iva Matolínová, Vladimír Matolín, Martin Janata, Tereza Bělinová, Marie Hubálek Kalbáčová

**Affiliations:** Department of Surface and Plasma Science, Faculty of Mathematics and Physics, Charles University V Holešovičkách 2 18000 Prague Czech Republic xiaohui.ju@mff.cuni.cz; Department of Chemical Physics and Optics, Faculty of Mathematics and Physics, Charles University Ke Karlovu 3 12116 Prague Czech Republic; Biomedical Center, Medical Faculty in Pilsen, Charles University Alej Svobody 1655/76 32300 Pilsen Czech Republic; Institute of Pathological Physiology, 1st Faculty of Medicine, Charles University U Nemocnice 5 12853 Prague Czech Republic

## Abstract

One of the biggest challenges for the biomedical applications of cerium oxide nanoparticles (CeNPs) is to maintain their colloidal stability and catalytic activity as enzyme mimetics after nanoparticles enter the human cellular environment. This work examines the influences of CeNP surface properties on their colloidal stability and catalytic activity in cell culture media (CCM). Near-spherical CeNPs stabilized *via* different hydrophilic polymers were prepared through a wet-chemical precipitation method. CeNPs were stabilized *via* either electrostatic forces, steric forces, or a combination of both, generated by surface functionalization. CeNPs with electrostatic stabilization adsorb more proteins compared to CeNPs with only steric stabilization. The protein coverage further improves CeNPs colloidal stability in CCM. CeNPs with steric polymer stabilizations exhibited better resistance against agglomeration caused by the high ionic strength in CCM. These results suggest a strong correlation between CeNPs intrinsic surface properties and the extrinsic influences of the environment. The most stabilized sample in CCM is poly(acrylic acid) coated CeNPs (PAA-CeNPs), with a combination of both electrostatic and steric forces on the surface. It shows a hydrodynamic diameter of 15 nm while preserving 90% of its antioxidant activity in CCM. PAA-CeNPs are non-toxic to the osteoblastic cell line SAOS-2 and exhibit promising potential as a therapeutic alternative.

## Abstract 2

Cerium oxide nanoparticles coated with poly(acrylic acid) exhibited high colloidal stability and preserved catalytic activity in cell culture media.

## Graphical abstract

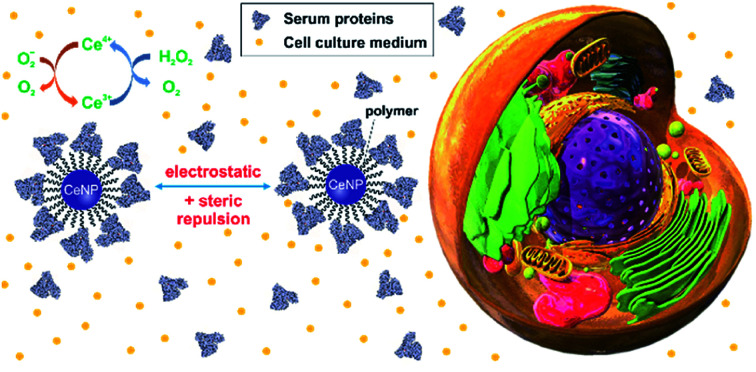

## Introduction

Cerium oxide nanoparticles (CeNPs) possessing catalytic activities to scavenge reactive oxygen species (ROS) have received much attention for their potential application as nanomedicine.^1–3^ Initial studies show that CeNPs can function like superoxide dismutase (SOD), catalase, and oxidase with cell-protective, neuro-protective, and cardio-protective effects in the treatment of several diseases.^4–6^ The enzyme mimetic behaviour of CeNPs is attributed to their oxygen storage capacity: cerium oxide can undergo reduction/oxidation cycles through electron charge transfer between Ce^3+^ and Ce^4+^ by capturing, storing, or releasing oxygen on their surfaces.^5–7^ For this reason, the surface properties of CeNPs play critical roles in the performance of modulating concentrations of ROS in cellular environments.

On the one hand, CeNPs surfaces are often functionalized during synthesis in order to avoid nanoparticles (NPs) agglomeration in the human cellular environment.^8–13^ Many polymers, such as dextran, chitosan, poly(acrylic acid) (PAA), and oleic acid, have been reported to act as good coating agents to improve CeNPs colloidal stability and dispersity.^14–16^ Thinner layer of polymers is reported to preserve CeNPs catalytic activities without blocking the electron charge transfer pathway on the nanoparticle's surface.^17^ On the other hand, when entering extracellular fluids, CeNPs are subjected to a range of extrinsic forces that further modify their surfaces and determine their behaviour before cellular uptake.^18^ NPs interaction with cell culture media (CCM) during *in vitro* tests causes irreversible aggregation of NPs, thus significantly increase their overall cytotoxicity and further causes mechanical stress to the cells.^19^ The final characteristics of CeNPs (“what the cell sees”) can be very different from what was initially produced in the laboratory, further causing discrepancies between reports of cell responses to CeNPs.^13^

Biological solutions, such as extracellular fluids or cell culture media, are complex systems containing electrolytes, proteins, lipids, vitamins, and other compounds. It helps the cells to maintain pH and osmolality as well as supplies them with necessary nutrients and growth factors.^13,20^ pH is one of the factors influencing CeNPs antioxidant behaviour during NPs–cell interaction. Although the cytosolic pH of most cells remains near neutral, the subcellular organelles can be acidic or basic over a broad range of pH.^21^ CeNPs exhibit more oxidase-like and toxic activities at the acidic environment, due to the dissolution of Ce ions into acidic media.^12^ The high content of electrolyte components is another factor influencing CeNPs colloidal behaviour and catalytic activity in CCM.^20^ The high ionic strength of CCM not only can unbalance the intermolecular and surface forces that stabilize CeNPs colloidal solution,^20^ but it can also introduce a specific “poisoning effect” for CeNPs catalytic sites.^22^ Seal and his co-workers showed that with the increase of the phosphate concentration in the aqueous solution, CeNPs catalytic activity as enzyme mimetics were decreased, due to the phosphate effect of inhibiting oxygen capture and release on CeNPs surfaces.^22–24^ The third factor to consider interacting with CeNPs in CCM is protein corona formation.^20^ The proteins in the biological system (plasma in blood or serum in CCM) are the first entities that CeNPs interact with when entering the biological system. The NPs–protein complex is a new entity with a biomolecular corona being the interface between the NPs and the cellular system.^20,25,26^ Protein has been reported to either stabilize NPs through adsorption onto particle surfaces or destabilize NPs through a competitive exchange of stabilizing molecules.^20^ Several nanomaterials have been shown to aggregate under serum-rich conditions, rendering their biological applications.^19,25,27–29^ Preferential protein adsorption of CeNPs has been reported to depend either on their surface charges (zeta potentials)^30^ or hydrophobicity.^25^ Recent research reported that the protein corona formation on the surface of nanodiamonds depends on the NPs electrostatic interaction with the protein, rather than the hydrophobicity of NPs.^29^ In the abovementioned cases, the surface functional groups play a significant role in determining protein binding affinities in NPs surface.^31^ The factors influencing the interactions between CeNPs and proteins in CCM remains unclear.

In this research, we investigated individual effect of intrinsic and extrinsic properties on CeNPs colloidal stability and catalytic activity as enzyme mimetics in CCM. We synthesized CeNPs without coating as well CeNPs with different hydrophilic polymer coatings through a chemical precipitation method. The physicochemical properties of these CeNPs were characterized *via* several analytical tools. The effect of the extrinsic properties was monitored by measuring CeNPs colloidal stability, H_2_O_2_ scavenging activities, SOD and catalase mimetic activities, and quantification of CeNPs protein adsorption in water and CCM. The *in vitro* biocompatibility of CeNPs was tested with osteoblastic cell line (SAOS-2). Last, the interaction of CeNPs with the cellular matrix in CCM prior to cellular uptake was investigated by microscopic method.

## Materials and methods

### Synthesis of non-coated and polymer-coated CeNPs samples

The CeNPs solutions were prepared by a wet-chemical precipitation method, as described previously.^12,19^ Briefly, cerium(iii) nitrate hexahydrate (Ce(NO_3_)_3_·6H_2_O) (Sigma Aldrich, 99.999%) was dissolved in water. For polymer-coated CeNPs, either 0.5% (final weight percentage concentration) of poly(acrylic acid) (molecular weight around 1200, Sigma Aldrich) or dextran (molecular weight around 6000, Sigma Aldrich) were added together with cerium(iii) nitrate hexahydrate solution and mixed thoroughly. Ammonium hydroxide (30%, Sigma Aldrich) was added to the mixture dropwise under continuous stirring for 24 h at room temperature. The preparation was then centrifuged at 4000 rpm for two 30 min cycles to settle down any debris and large agglomerates. The supernatant solution was purified using an Amicon cell with a MWCO 30k cut-off membrane (Millipore Inc.) until the final pH reached 7. Synthesized bare CeNPs without polymers (Syn-CeNPs) were centrifuged at 4000 rpm for two 30 min cycles, while dextran and PAA coated CeNPs (Dex-CeNPs and PAA-CeNPs) were further centrifuged at 13 000 rpm for another two 30 min cycles to remove debris introduced through filtration. Commercially available Com-CeNPs dispersion in 4% methoxyacetic acid solution (20%, low pH, <5.0 nm APS number weighted, Alfa Aesar) was used in this study to compare with lab-synthesized CeNPs solutions. All CeNPs solutions were diluted to 1 mg ml^−1^ (final concentration) for further tests unless otherwise mentioned.

### Nanoparticles characterization

Samples were prepared as described above. Transmission Electron Microscope (TEM) was performed using a JEOL NEOARM 200 F microscope with a Schottky-type field emission gun at an accelerating voltage of 200 kV with a resolution of 100 pm in HRTEM mode. The microscope was equipped with a TVIPS XF416 CMOS camera. The TEM samples were prepared by diluting CeNPs to a proper concentration and then drop-casting onto a holey carbon-coated copper 300-mesh grid (Agar Scientific) followed by air-dry.

Atomic Force Microscope (AFM) images were collected with a Nanowizard 3 (JPK Instruments, Berlin, Germany) combined with an IX73 microscope (Olympus, Melville, NY). The samples have been diluted to proper concentration and then drop-casted on a mica surface and dried out and measured immediately. For the measurement, an ACTA cantilever was used in AC AFM mode. The pictures were analysed to get the distribution of nanoparticles sizes; for this purpose, the Mathematica software was used to give the height of each nanoparticle.

Dynamic Light Scattering (DLS) measurement was carried out by using Zetasizer Pro (Malvern Co.). Hydrodynamic diameter sizes (*D*_H_) and zeta potential (*Z*_P_) were measured using samples as prepared, and *D*_H_ was expressed as volume-weighted. Zeta potential titration measurement was carried out by the addition of an auto titrator with a degasser attached to the Zetasizer. Hydrochloric acid (0.25 mol l^−1^ and 0.025 mol l^−1^) and sodium hydroxide (0.25 mol l^−1^) were used for the auto-titration in the pH range of 8 to 2 (from base to acid). When measuring *D*_H_ and *Z*_P_ for CeNPs in various solutions, the CeNPs were incubated with the solutions at room temperature for at least two hours to reach stabilization before the test (at the final concentration of 1 mg ml^−1^). The conductivity of all samples was monitored and maintained at the same level (0.25 ± 0.05 mS cm^−1^) during DLS measurement.

### X-ray photoelectron spectroscopy (XPS) characterization

CeNPs solutions were dried at 50 °C in air for 72 hours and ground into fine powders. Powdered samples were pressed on a 0.2 mm thick indium foil (99.99%, Lesker) and mounted on a sample holder for XPS characterization. To decrease the charging effect, all samples were measured in 1 mbar of argon (purity of 99.9999%) at a near-ambient pressure X-ray photoelectron spectroscopy (NAP-XPS) station. The NAP-XPS set-up (Specs Surface Nano Analysis, GmbH) was equipped with a monochromatized Al Kα X-ray source (1486.6 eV) and a multichannel electron energy analyser (Specs Phoibos 150) coupled with a differentially pumped electrostatic pre-lens system. XPS Ce 3d, C 1s, and O 1s core-level spectra were collected for all samples. The data were analysed using KolXPD fitting software.^32^ All photoemission spectra were fitted with a mixture of Lorentzian and Gaussian function profiles after Shirley background subtraction. The measured core-level spectra of Ce 3d were fitted with a total of five doublets corresponding to the Ce^3+^ and Ce^4+^ states to evaluate the oxidation state of cerium in all samples.^33^ Spectral lines were broadened due to the charging effect. However, the fitting criteria were used in the same way for all the measured data; thus, the calculation of Ce^3+^ percentage (Ce^3+^/(Ce^3+^ + Ce^4+^)) is comparable across all samples.

### Protein adsorption quantification

To characterize protein adsorption, 1 ml of 2 mg ml^−1^ of CeNPs solutions were mixed with 1 ml of 2 mg protein per ml BSA (Sigma Aldrich) dissolved in water, or 2 mg protein per ml FBS in DMEM. The mixture was vortexed vigorously and set at room temperature for two hours. CeNPs were then centrifuged, and the concentration of BSA or FBS was determined in the supernatant using a Multiskan GO UV-visible spectrophotometer microplate reader (Thermo Fisher Scientific). Bradford reagent (Sigma Aldrich) was used for protein quantification by measuring absorbance at 595 nm wavelength. The calibration curve was prepared using a series of known concentrations of BSA. The protein adsorbed onto CeNPs was quantified by the differences between initial protein concentration in the mixture and the protein in the supernatant after centrifugation.

### CeNPs antioxidant activity measurement

UV-visible spectra of CeNPs solutions were obtained using the Multiskan GO UV-visible spectrophotometer with a microplate reader (Thermo Fisher Scientific) with absorbance from 200 nm to 800 nm at 1 nm per step. The H_2_O_2_ scavenging activities were measured by adding 10 μmol H_2_O_2_ (Sigma Aldrich) to 1 mg ml^−1^ CeNPs solutions followed by vigorous mixing. The H_2_O_2_ scavenging activity was defined as Δ*λ*, the wavelength shifts when the absorbance value is at 0.3, as established in ref. 17.

### SOD and catalase activities

SOD and catalase activities of CeNPs were measured by enzyme assay kit (SOD Assay Kit, catalogue no. 19160; Catalase Assay Kit, catalogue no. CAT100, Sigma Aldrich) following manufacturer's instructions. These assays were used to quantify the preserved SOD and catalase activities of tested CeNPs in water and after CeNPs incubation with different media conditions.

For SOD activity assay, CeNPs were diluted to proper concentration to make sure measured SOD reach to a linear range of enzyme inhibition rate between 20 to 80%. Additional negative controls were added to account for the possible interference from the used media. Formazan dye production was determined by measuring absorbance at 440 nm after 20 min reaction by the Multiskan GO UV-visible spectrophotometer equipped with a microplate reader (Thermo Fisher Scientific).

For catalase activity assay, the colorimetric method uses a substituted phenol (3,5-dichloro-2-hydroxybenzenesulfonic acid), which couples with 4-amino antipyrine in the presence of hydrogen peroxide and horseradish peroxidase (HRP) to give a red quinoneimine dye (*N*-(4-antipyryl)-3-chloro-5-sulfonate-benzoquinone-monoimine) that absorbs at 520 nm. The standard curve of H_2_O_2_ concentration was measured freshly for each sample measurement. CeNPs incubated in different media were diluted to the total protein concentration under 50 mg ml^−1^ and glucose concentration under 5 mM to avoid interference of the assay.

### Cell cultivation and cytotoxicity test

Human osteoblastic cells (SAOS-2, DSMZ, Germany) were cultivated over the long-term in standard McCoy's 5A medium (GSE Healthcare – HyClone) supplemented with 15% of FBS (Biosera), l-glutamine (Life Technologies), 10 000 U ml^−1^ of penicillin and 10 μg ml^−1^ of streptomycin (both Sigma Aldrich) in a CO_2_ humidified incubator at 37 °C.

For cytotoxicity testing, the osteoblastic cells were seeded in the standard cultivation medium at a concentration of 10 000 cells per cm^2^ on 96-well plates and incubated for 24 h. The cells were then washed in phosphate buffer saline (PBS, Thermo Fisher Scientific – Gibco), followed by the addition of the appropriate CeNPs-containing medium. All the tested CeNPs were mixed either in Dulbecco's Modified Eagle Medium (DMEM, Thermo Fisher Scientific – Gibco) supplemented with 5% of FBS, or in the non-FBS-supplemented DMEM and added to cells for six hours. Then FBS to a final concentration of 5% was added to all samples (to avoid cell inhibition and death caused by a lack of nutrients) and incubated for 24 or 72 h. The medium containing the nanoparticles was discarded at each time point, and a fresh medium with 10% of tetrazolium stain (MTS, Cell Titer 961 AqueousOne, Promega) was added to the cells, which were then incubated for two hours in a CO_2_ incubator. The optical density was measured using a microplate reader (Multiskan MS) at 492 nm, subtracting the background at 620 nm. The values obtained were related to the corresponding controls (non-treated cells) in terms of percentage. The results obtained were statistically evaluated using the Statistica program (extreme and remote value subtraction based on box graphs, the non-parametric Wilcoxon matched-pairs test).

### Characterization of cell–CeNPs interaction in CCM

For Scanning Electron Microscope (SEM) measurement purposes, SAOS-2 cells were pre-cultivated on the 1 × 1 cm^2^ Si-wafer slides in a 24-well plate in the standard cultivation medium for 24 h at a concentration of 10 000 cells per cm^2^. The cells were then washed in PBS, and DMEM supplemented with 5% of FBS to which 100 μg ml^−1^ of CeNPs was added for 24 hours. The cells were then washed in PBS and fixed in 2.5% glutaraldehyde (Sigma Aldrich) in PBS at 4 °C overnight. Following fixation, the samples were dehydrated *via* an ethanol row (10 min, room temperature, in 20%, 40%, 50%, 60%, 70%, 100% ethanol, acetone), dried using a critical point dryer (Bal-Tec CPD 030), and observed using a field-emission SEM (Tescan Mira 3) equipped with an energy-dispersive X-ray (EDX) detector (XFlash, Bruker) at a primary electron energy of 15 keV.

## Results and discussion

### Synthesis and characterization of CeNPs in the solution

Four kinds of CeNPs representing selective functionalization mechanisms were investigated in this work. Near-spherical CeNPs with crystallite diameters ranging from 2 to 6 nm were synthesized with or without the presence of hydrophilic polymers. Synthesized CeNPs without any coatings were named as Syn-CeNPs. Dextran and PAA were added during the precipitation process, respectively, resulting in two polymer-coated CeNPs (named as Dex-CeNPs and PAA-CeNPs). Commercially available CeNPs in a 4% methoxyacetic acid solution (named as Com-CeNPs) were used for comparison.

**Fig. 1:**
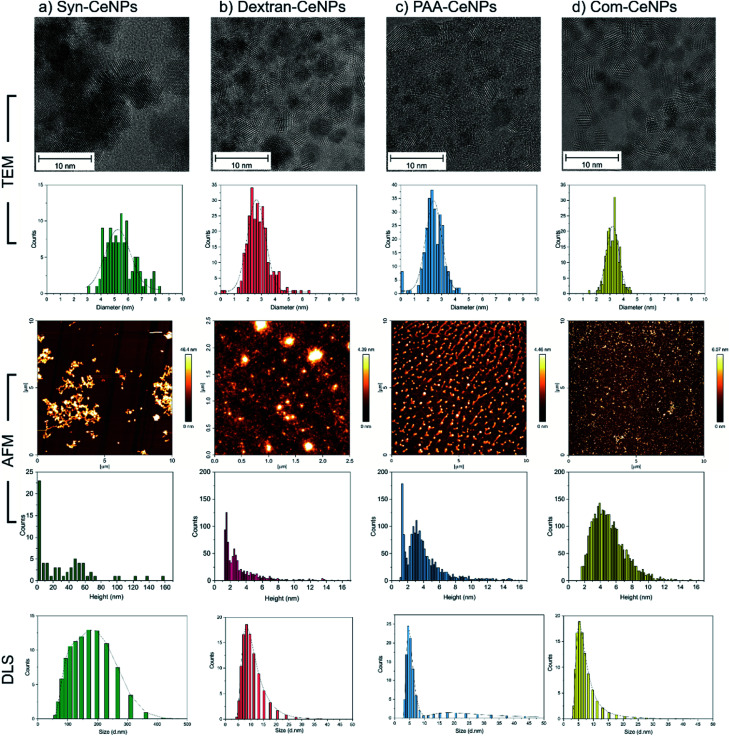
Size measurement of CeNPs by TEM (micrographs and corresponding diameter distribution histograms), height measurement by AFM (micrographs and corresponding height distribution histograms) and hydrodynamic diameter distribution by DLS: (a) Syn-CeNPs; (b) Dex-CeNPs; (c) PAA-CeNPs; and (d) Com-CeNPs. Samples from (a) to (c) were prepared in water. Sample (d) was received in methoxyacetic acid solution and further diluted by water. All samples were diluted to 1 mg ml^−1^ before characterization.

TEM and AFM were used to characterize the sizes of CeNPs samples investigated in this work. The synthesis with the presence of polymers (dextran and PAA) yielded the smallest CeNPs crystals, with crystal sizes of 2.8 ± 0.8 nm for Dex-CeNPs and 2.4 ± 0.7 nm for PAA-CeNPs, as observed by TEM (Fig. 1). Without polymers, Syn-CeNPs nanocrystals growth was promoted up to 5.5 ± 1.1 nm. Com-CeNPs exhibited an average size distribution of 3.2 ± 0.5 nm, corresponding to the size of less than 5 nm indicated by the manufacturer. In the case of the three laboratory-prepared CeNPs, peak height under 1.5 nm was observed by the AFM in Fig. 1. These peaks represented the dried surfactants during sample preparation. Besides the surfactant effect, the height of all samples measured by AFM corresponds to the crystalline sizes measured by TEM, except Syn-CeNPs. Syn-CeNPs exhibited a broad height distribution range up to 200 nm, with the main peak around 50 nm. These results suggested that Syn-CeNPs had a certain degree of agglomeration in water solution compared with the other three kinds of CeNPs. The DLS method was used to characterize the hydrodynamic diameters (*D*_H_) distributions of prepared CeNPs in aqueous solutions. The *D*_H_ value of Syn-CeNPs in the range of 176.0 ± 2.0 nm (Fig. 1) further confirmed CeNPs aggregation, as also observed by AFM. For the other three samples of Dex-CeNPs, PAA-CeNPs, and Com-CeNPs, the *D*_H_ values were 9.5 ± 0.7 nm, 10.4 ± 0.3 nm, and 6.6 ± 0.4 nm, respectively, indicating a good dispersity in their aqueous solution.

**Fig. 2:**
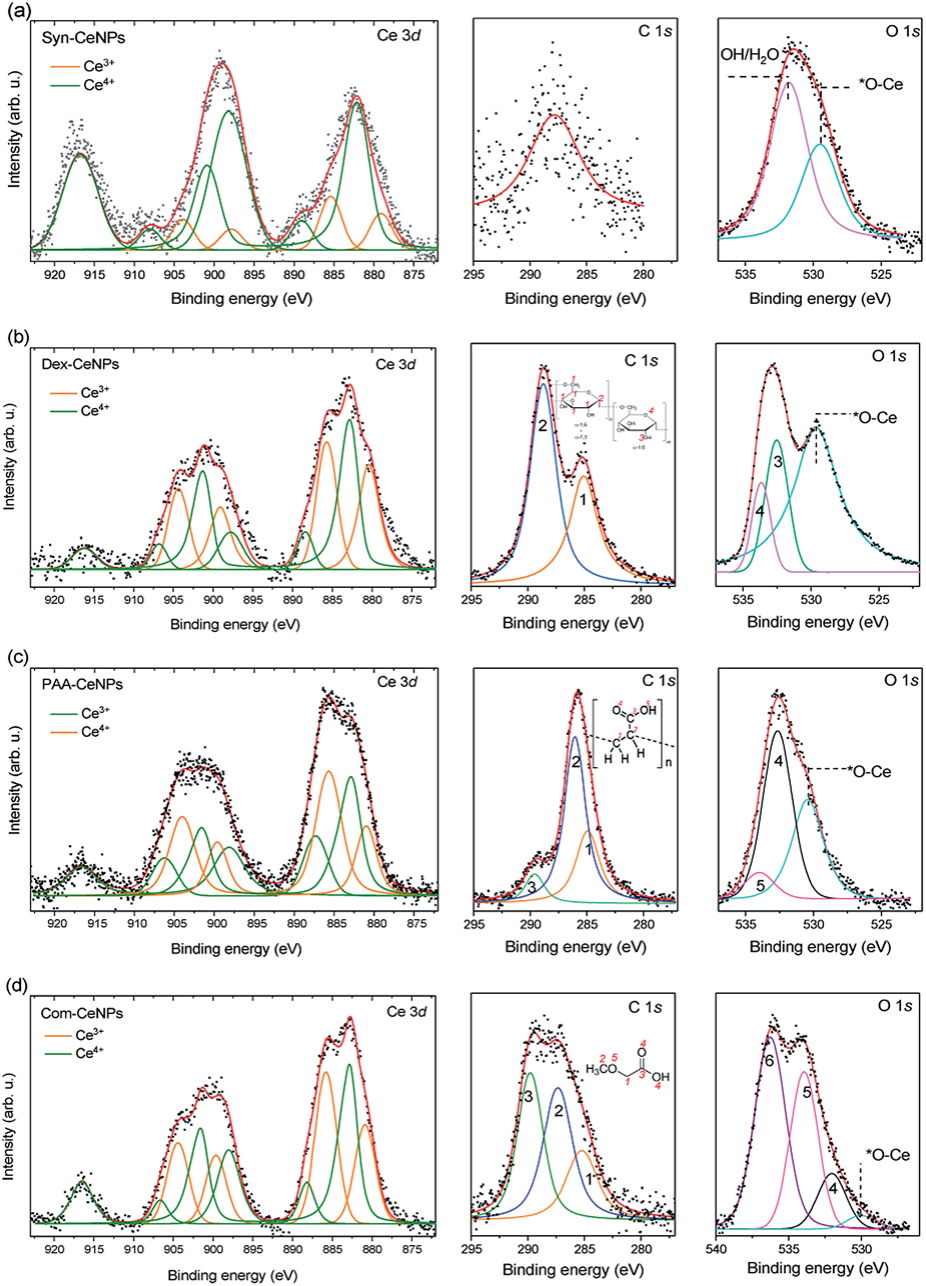
XPS Ce 3d, C 1s, and O 1s spectra of CeNPs: (a) Syn-CeNPs; (b) Dex-CeNPs; (c) PAA-CeNPs; and (d) Com-CeNPs. The fits in Ce 3d spectra represent Ce^3+^ (orange line) and Ce^4+^ (green line) oxidation states, respectively.

XPS was used to characterize the surface chemical properties of CeNPs, as shown in Fig. 2. The Ce^3+^ percentage is similar within Com-CeNPs (47%), Dex-CeNPs (50%), and PAA-CeNPs (46%). Syn-CeNPs has a lower percentage of Ce^3+^ (16%) compared with the other three samples. This finding corresponds to the trend of their measured crystalline sizes by TEM and AFM, where smaller CeNPs tended to have higher Ce^3+^ concentrations on its surface.^6,15,34^ The C 1s spectrum of Syn-CeNPs (Fig. 2a) suggests that there was no carbon presence. The peak at ∼289.7 eV was attributed to cerium oxide (Ce 4s). The O 1s spectrum of Syn-CeNPs consisted of one peak from cerium oxide (∼529.5 eV), and another peak from hydroxyl groups adsorbed on ceria (531.7 eV).^33^ No coating compound was observed for Syn-CeNPs. The C 1s spectrum of Dex-CeNPs (Fig. 2b) was fitted with carbon peaks at 285.0 eV and 288.6 eV originated from the bonds of C̲–C/H and O–C̲–O in dextran.^35^ However, the typical peak of C–O from dextran around 287 eV was not observed. It is suspected that C–OH groups were interconnected with Ce; thus, such a peak is not observable from the XPS spectra. The peaks at O 1s spectrum of Dex-CeNPs consisted of the contribution of lattice oxygen from cerium oxide (529.5 eV) and contribution from dextran (533.0 and 533.6 eV). The C 1s spectrum of PAA-CeNPs (Fig. 2c) contained carbon peaks around 285, 286, and 290 eV, which were attributed to C̲–H/C̲–C, C̲–COOH, and C–C̲=O, respectively. PAA-CeNPs O 1s spectrum consisted of a peak from cerium oxide (529.5 eV), and two peaks (the peak at 532.7 eV was assigned to O̲=C–OH (4), and the peak at 534.1 eV was assigned to O=C–O̲H (5)) associated with the structure of PAA.^36,37^ The C 1s spectrum of Com-CeNPs (Fig. 2d) was fitted with three peaks around 285, 287, and 290 eV, which were assigned to C̲–C/C–H, C̲–O, and HO–C̲=O, respectively.^35,37^ The O 1s spectrum was fitted by four peaks. The peak at 529.5 eV was attributed to the lattice oxygen from cerium oxide. Peak 4 was attributed to the O̲H group. Peak 5 and 6 at 532 eV and 534 eV corresponded to the bonds of O̲=C–H and O̲=C–C, respectively. The C 1s and O 1s spectra of Com-CeNPs suggested the presence of methoxyacetic acid on the CeNPs surface since methoxyacetic acid was used in CeNPs solutions as indicated by the manufacturer.

**Fig. 3:**
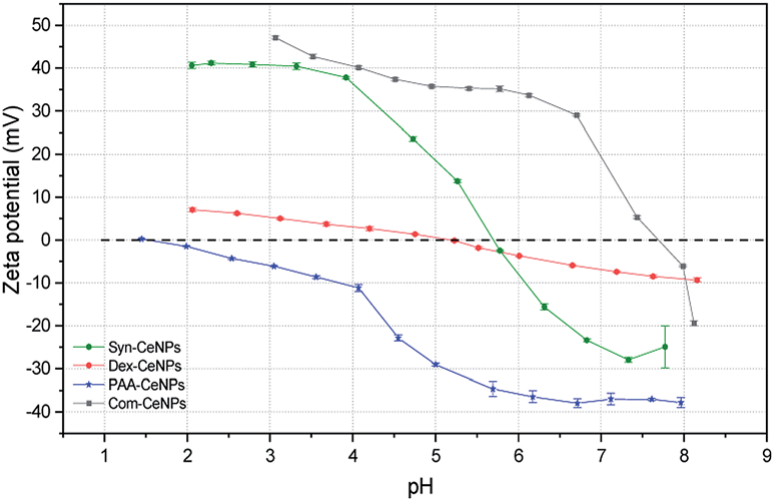
Zeta potential titration curve of the CeNPs samples as a function of the pH. The titrations were performed at the same level of conductivity, indicating the same ionic strength of the solutions during titration. The colloidal system is the least stable at the isoelectric point (IEP) when the *Z*_P_ is zero. There are no inter-particle repulsive forces due to the absence of the particle surface charges at the IEP.

The surface charges of prepared CeNPs samples were measured using the zeta potential (*Z*_P_) analysis. Syn-CeNPs and PAA-CeNPs showed negative *Z*_P_ values of −28 mV and −48 mV, respectively, in contrast to Com-CeNPs, which were positively charged at 26 mV. The *Z*_P_ value of Dex-CeNPs was close to zero (−7 mV), indicating a neutral charge of the polymers. Since these three laboratory-synthesized samples were measured in a water solution with pH near neutral while the Com-CeNPs were measured in its original acidic solution, we further evaluated their colloidal stability with the variation of pH during titration (Fig. 3). The correlation of *Z*_P_ to pH offers a reference to interpret how the varying pH may affect the surface charge of the CeNPs, and, therefore, their colloidal stability. The pH of extracellular fluid and cellular cytoplasm is typically about 7.2–7.4.^21^ The Com-CeNPs sample was the least stable one at this pH range since its *Z*_P_ value was very close to its isoelectric point (IEP = 7.7); it tends to agglomerate at such pH ranges. On the other hand, the PAA-CeNPs sample was the most stable one since the absolute value of the *Z*_P_ at pH 7.2 was above 30 mV. However, when interpreting the titration curves, it should be noted that the effect of pH titration on *Z*_P_ is a combination of both ionic strength changes in aqueous solution and pH itself since the titration solvents contain Na^+^ and Cl^−^ at a maximum concentration of 0.25 mol l^−1^.

According to the Derjaguin–Landau–Verwey–Overbeek (DLVO) theory, colloidal stability is ensured when steric and electrostatic interactions can counterbalance the short-range van der Waals attractive interactions.^38^ The Syn-CeNPs sample was charged as a consequence of hydroxyl groups dissociated from water adsorbing on the oxide surface, as pointed out by Mullins.^33^ A large portion of counterions clouds was formed around the particles, extending far from the NPs surface due to the low ionic strength of the original water solution. This counterion clouds further repel particle–particle interaction originated as electrostatic forces.^38^ Polymers such as dextran and PAA adsorbed on the surface of nanoparticles, further preventing CeNPs interaction by creating steric repulsion on their surface.^38^ The close-to-zero *Z*_P_ value of Dex-CeNPs indicated no attracted counterions on the CeNPs surface due to the neutral charges of the polymer itself.^20,38^ PAA-CeNPs exhibited a value of negative *Z*_P_ due to the charged polymer (PAA) adsorbed on the CeNPs surface.^27,39^ While strongly adsorbed polymers onto CeNPs surface can act as a physical barrier against aggregation, charged polymers provide extra electrosteric repulsion (a combination of electrostatic and steric forces)^20^ between individual NPs. As-received Com-CeNPs had negatively charged carboxyl groups on their surface from the methoxyacetic acid solution (shown as positive *Z*_P_), thus generating electrostatic repulsion in-between NPs. Based on the abovementioned discussion, the mechanism of CeNPs stabilization in aqueous solution and their characterization parameters are summarized in Table 1. The role of such stabilization mechanisms in determining CeNPs behaviour in CCM is further discussed in the following section.

**Table 1:** Summary of CeNPs physicochemical properties. Samples were all prepared as CeNPs dispersed in water with no electrolytes, except the sample Com-CeNPs dispersed in methoxyacetic acid solution. All samples were diluted to 1 mg ml^−1^ before characterization

Properties	Syn-CeNPs	Dex-CeNPs	PAA-CeNPs	Com-CeNPs
Size by TEM (nm)	5.5 ± 1.1	2.8 ± 0.8	2.4 ± 0.7	3.2 ± 0.5
Height by AFM (nm)	50.2 ± 0.9	2.3 ± 0.1	3.0 ± 0.5	3.4 ± 0.5
*D*_H_ (nm)	176.0 ± 2.0	9.5 ± 0.7	10.4 ± 0.3	6.6 ± 0.4
*Z*_P_ (mV)	−28.0 ± 1.2	−6.5 ± 0.1	−47.9 ± 0.9	26.0 ± 0.6
Ce^3+^ (%)	16	50	46	47
Stabilizing mechanism	Electrostatic	Steric	Electrostatic + steric	Electrostatic

### Protein–CeNPs interactions and their colloidal stability in CCM

When the influence of physicochemical properties of CeNPs on cells is evaluated *in vitro*, CeNPs interact with the components of the present CCM (basic medium with supplements) before any cellular contact.^20^ In the CCM, the primary source of protein comes from its supplement – serum, a liquid part of blood plasma providing vital elements for cell survival in culture (*e.g.*, growth factors, vitamins, lipids, hormones, *etc.*). It consists of around 10 000 proteins, of which only a small fraction is highly abundant (*e.g.*, albumin, immunoglobulin G, fibrinogen, apolipoproteins, and complement factors). Fetal bovine serum (FBS) is widely added as a growth supplement for the cultivation of various mammalian cell lines.^40^ FBS is the liquid fraction of clotted blood from fetal calves, containing a large number of nutritional and macromolecular factors essential for cell growth, and the primary and most abundant component is bovine serum albumin (BSA).^40,41^ Furthermore, BSA is 98% similar to human analogue while being more widely studied.^42^ Here we chose to use both BSA (as a representative of a single broadly studied protein) and FBS (as a representative of broadly studied cell cultivation supplement containing a mixture of different proteins) to study the CeNPs and protein interactions.

**Fig. 4:**
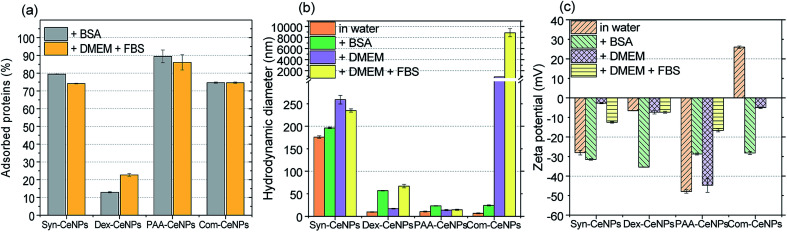
(a) The amount of BSA from water and the amount of FBS from DMEM solutions adsorbed onto CeNPs. The same amount of protein (1 mg ml^−1^) was added during the adsorption experiment. (b) Hydrodynamic diameter and (c) zeta potential of CeNPs in different solutions: water, 0.1% BSA solution, DMEM, and DMEM with FBS addition.

Fig. 4a shows that Syn-CeNPs, PAA-CeNPs, and Com-CeNPs exhibited a high amount of protein adsorption (more than 70%), regardless of the protein supplement types. On the contrary, the amount of proteins adsorbed onto Dex-CeNPs was relatively low (less than 30%). The *D*_H_ increased after BSA adsorption from water by about 15 to 30 nm for Syn-CeNPs, PAA-CeNPs, and Com-CeNPs (Fig. 4b). Such an increase in the three CeNPs was highly unlikely to be caused by aggregation.^19,27,43^ This increase in size could only be attributed to proteins adsorbed on the NPs surfaces, which corresponds to the observed higher protein adsorption (Fig. 4a). After BSA co-incubation with CeNPs, the *Z*_P_ of all CeNPs showed a similar negative value (Fig. 4c), indicating sufficient protein coverage of the CeNPs surface. These results are in agreement with the higher protein adsorption observed above. As for the case of Dex-CeNPs, the amount of adsorbed proteins was less than half of the amount on the other three CeNPs, while the *D*_H_ increased from 10 to 57 nm. Such an effect will be further discussed later. The above-presented results indicate that CeNPs stabilized electrostatically behave differently in BSA solution compared with CeNPs without electrostatic stabilization (Dex-CeNPs).

The medium for mammalian cell cultivation, taking Dulbecco's Modified Eagle Medium (DMEM), for example, contains a high amount of inorganic salts (Table S1†). We further examined the effect of culture media compounds (DMEM only) on CeNPs colloidal stability when proteins were not supplemented. For Syn-CeNPs, *D*_H_ increased from 176 nm to 260 nm after incubation in DMEM (Fig. 4b). A significant increase in *D*_H_ from 7 nm to 883 nm for Com-CeNPs indicated a more severe particle agglomeration. The *Z*_P_ values of Syn-CeNPs became closer to zero (Fig. 4c). For Dex-CeNPs and PAA-CeNPs, their sizes were only increased by 4–7 nm compared to their *D*_H_ in water, most probably due to the cations attracted to the surface.^27^ It proves that the steric polymer coatings of dextran and PAA make CeNPs more resistant to the media changes from water to DMEM.

When 5% of FBS was added into DMEM, the effect of supplemental protein and culture media were evaluated at the same time. The amount of adsorbed proteins and the value of *D*_H_ for Syn-CeNPs remained almost unchanged (Fig. 4). It is suspected that proteins or amino acids exchanged with cations and anions adsorbed onto the Syn-CeNPs surface. It leads to a slight modification of the surface properties, as indicated by the observed changes in *Z*_P_ from −3 mV to −13 mV for Syn-CeNPs after protein adsorption (Fig. 4c). For Dex-CeNPs, the increase of *D*_H_ was due to the combined effect of DMEM and FBS, increasing from 17 nm to 67 nm after the addition of FBS to the DMEM, which is also observed as the effect of BSA protein adsorption itself. For PAA-CeNPs, the *D*_H_ change in DMEM with FBS was also not significant compared with *D*_H_ in water. *Z*_P_ values of PAA-CeNPs after co-incubation of DMEM with FBS was changed from −45 mV to −17 mV (Fig. 4c), which is caused by protein/amino acid adsorption on its surface. For Com-CeNPs incubated with DMEM and FBS, particle sedimentation was further observed, as in the case of DMEM.

**Scheme 1:**
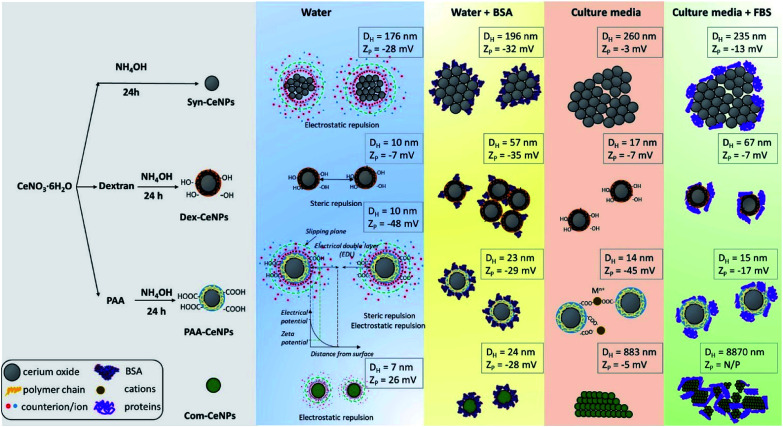
Schematic representation of CeNPs synthesis flowchart, their colloidal stabilization mechanism in various solvents, and their hydrodynamic diameter as well as zeta potentials.

In Scheme 1, we summarized the colloidal behaviour of prepared CeNPs, considering the surface functionalization, media effect, protein adsorption, and ion influence from CCM. When incubating together with CCM, CeNPs are covered by proteins, resulting in CeNPs surface decoration, also referred to as a protein corona formation on the particle surface.^26^ The surface properties of CeNPs has detrimental effects on the protein adsorption process.^19,30^ Although positively charged NPs have been reported to favour the protein adsorption,^19,30^ we observed high protein adsorption even for two negatively charged samples (Syn-CeNPs and PAA-CeNPs). We did not observe a positive correlation between NPs surface zeta potential and the amount of protein adsorption. We suspect that electrostatic interaction is the dominant force for CeNPs to adsorb proteins in aqueous solutions, regardless of surface coatings and surface charges, as reported previously.^29,44,45^ Protein flocculation might cause apparent aggregation. The term flocculation is used here for the formation of slightly loose aggregates of particles linked together by a polymer or protein. It is distinct from coagulation in which the particles come into close contact as a result of changes in the electrical double layer (EDL) around the particles.^38^ In the case of Dex-CeNPs, proteins may attach to the dextran surface at several points, but for some of its length, protein can extend into the solution. Due to the reactivity of both carboxyl (–COOH) and amino (–NH_3_) end of the amino acids interacting with the extensive hydroxyl (–OH) groups of dextran surfaces, two Dex-CeNPs can be brought together. Thus the protein molecules can form a bridge between one particle and another, causing mild NPs aggregation.

When CeNPs were added to the CCM, their colloidal stability was influenced not only by the protein but also by the high ion content present in the media (Scheme 1). When switching the environment from water to CCM, the ionic strength of the solution is changed from low to high. The high ionic strength suppressed the EDL, and thus EDL repulsion was not sufficient enough as the stabilization forces. CeNPs were destabilized, and a greater extent of agglomeration was observed. Previous studies showed that electrostatically stabilized NPs have generally shown poor stability in CCM.^28,43,46^ Ionic strength can also competitively exchange stabilizing molecules with other media components.^20^ In our study, we observed agglomeration of Syn-CeNPs and Com-CeNPs in DMEM, both of which were assumed to be stabilized through electrostatic forces only. On the contrary, Dex-CeNPs and PAA-CeNPs were more resistant to ionic strength changes due to the steric repulsion mediated by coating polymers. The presence of proteins in CCM resulted in more complex and highly variable effects on CeNPs stability. The changes in the *Z*_P_ values of CeNPs in the presence of biological media suggested significant surface changes of all CeNPs presented in this study. These changes can be explained by reactions of CeNPs with medium and FBS components such as BSA, variously charged amino-acids, salts, and vitamins. These components may bind to the CeNPs surface non-specifically, and as a result, the measured *Z*_P_ is not of the bare CeNPs but the CeNPs embedded in complex matrix.

### CeNPs catalytic activity as enzyme mimetics in CCM

The protective effect of CeNPs on cells is attributed to metal ion's ability to switch between Ce^3+^ and Ce^4+^ oxidation states due to the ease of oxygen atoms extracted from or donated to the lattice on the surface.^7^ With polymer steric blockage and the protein corona formation on CeNPs surface, how these CeNPs preserve their electron charge transferability in CCM needs to be carefully examined.

**Fig. 5:**
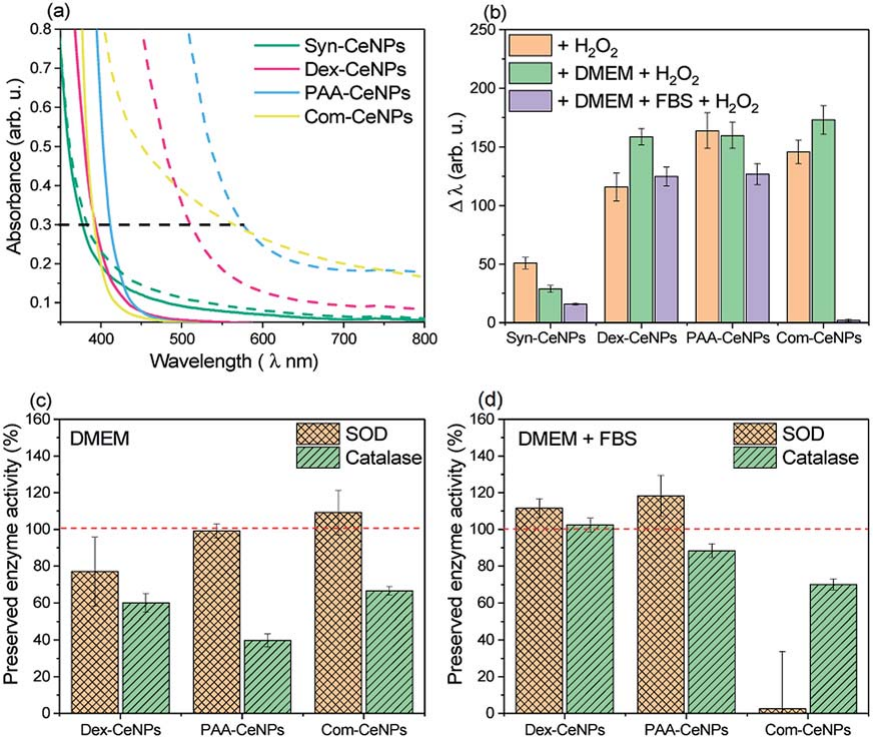
Antioxidant activities of CeNPs: (a) the adsorption spectra of CeNPs and their shift caused by the H_2_O_2_ quenching effect, measured in water by UV-vis. The red-shifted wavelength of the UV-visible band (Δ*λ*) was measured between the control (solid line) and the red-shifted band after H_2_O_2_ addition (dashed line) at the optical density of 0.3 (indicated by the horizontal black dashed line); (b) quantification of the measured red-shifted band (Δ*λ*) of CeNPs in different solutions with the addition of H_2_O_2_; (c) CeNPs SOD and catalase preserved activities due to the treatment of DMEM, using their activities measured in water solution as 100% control (red dashed line); (d) CeNPs SOD and catalase preserved activities due to the treatment of DMEM with 5% FBS supplement, using their activities measured in water solution as 100% control (red dashed line).

One established method to characterize the CeNPs catalytic activity and compare it across samples *ex situ* is to use colorimetric data to indicate the amount of Ce^4+^ produced.^17^ Colorimetric methods can monitor the antioxidant properties of CeNPs to scavenge H_2_O_2_ since the UV-visible absorption spectra of a CeNPs suspension gradually change when interacting with H_2_O_2_. As shown in Fig. 5a, the absorption spectra of CeNPs shifted after interacting with H_2_O_2_: the redshift (a change in absorbance to a longer wavelength)^17^ reflected the changes of Ce^3+^ to Ce^4+^. The shift (Δ*λ*) is defined by measuring the wavelength of 0.3 optical density before and after hydrogen peroxide addition.^17^Fig. 5b compared the changes of as-prepared CeNPs caused by hydrogen peroxide interaction under the condition in water, DMEM, or DMEM with 5% FBS addition. Note that the molecular weight of the CeNPs varies sensitively with their diameter, so only the equivalent particle concentration for all the NPs below 10 nm is comparable. Since Syn-CeNPs had exhibited much bigger agglomerates in solution than the other three CeNPs, and that antioxidant activity is substantially related to the sizes of the nanoparticles, the activity compared with the other three CeNPs tested in this study was not at the same level and thus was not taken into the discussion. The trend of CeNPs antioxidant activities in water followed the order: Com-CeNPs ≈ PAA-CeNPs > Dex-CeNPs. Research has indicated that the Ce^3+^ ratio is the most critical factor for CeNPs antioxidant activities.^15,17,23^ We presented three CeNPs with relatively similar Ce^3+^ ratio (47–50%) but coated with different thickness (Com < PAA < dextran, depending on molecular sizes). It shows that a lower-molecular-weight polymer preserved CeNPs catalytic activity better than higher-molecular-weight polymers. The thinner polymer had less influence on the electron charge transfer pathway of CeNPs without blocking its surface properties. In the presence of DMEM, antioxidant activities of all studied CeNPs were preserved. When FBS was added to the medium, Com-CeNPs lost almost all of their antioxidant activity. Such antioxidant activity loss was related to their poor colloidal stability in the medium as discussed previously.

We also examined the SOD and catalase enzyme mimetic activities of CeNPs when changing from water to DMEM. CeNPs exhibit SOD activities by converting superoxide anion O^2−^ to peroxide and oxygen; meanwhile the nanoparticle undergoes the transition of valance switch between Ce^3+^ and Ce^4+^.^5^ When acting as catalase, CeNPs possibly catalyse the reaction of decomposing H_2_O_2_ into O_2_ and H_2_O, although the role of Ce^3+^ in the catalytic mechanism remains unclear.^6^ In this study, we try to avoid comparing the absolute value of CeNPs catalytic activity due to the variation of polymer molecular weight of synthesized CeNPs. Instead, we focus on the comparison of preserved catalytic activity of these CeNPs after changing the incubation environment from water to different conditions of CCM. As can be seen in Fig. 5c, 80% of SOD activities were preserved in the presence of DMEM for all the tested CeNPs, while their catalase activities were reduced to 40–60% in DMEM. When FBS was added to the DMEM solution, 90% of the SOD and catalase activities were retained for Dex-CeNPs and PAA-CeNPs compared with their activities in the water. Com-CeNPs lost their SOD activity entirely, while with 70% preserved catalase activity in the case of DMEM with FBS. As we observed that Com-CeNPs formed sedimentation during interaction with FBS and DMEM, the colloidal system was interrupted, and they became questionable for further *in vitro* experiments.

Aside from the physicochemical properties of CeNPs, such as shapes and sizes, Ce^3+^ percentage, and coating polymers that are causing the differences in their catalytic activities, we believe that the extrinsic factors of ionic strength and protein interaction with CeNPs are highly underrated during *in vitro* test. Phosphate could poison CeNPs catalase and SOD responses by reacting with Ce^3+^ state and blocking their active sites, leading to CePO_4_ formation.^22,23^ However, such poisoning usually causes both enzyme mimetic activity reduction. We observed a similar trend for DMEM inhibition for CeNPs antioxidant activities, as reported previously, that the decrease of SOD activity was much smaller than that of catalase activity after phosphate buffer treatment. DMEM solution is a complicated system not only with a high presence of phosphate ions (∼1 mM) but also with a lot of amino acids and vitamins, which can cause profound poisoning to the CeNPs. While in the presence of FBS and DMEM, most of Dex-CeNPs and PAA-CeNPs enzyme activities were preserved, suggesting competitive adsorption of protein with surface component adsorbed from DMEM. Protein adsorption further minimized the ion poisoning effect caused by pure DMEM incubation with CeNPs.

### Interaction of CeNPs with human cells and their impact on cell metabolic activity

It has been well established that CeNPs, as compared to other metal oxides NPs, are non-toxic to mammalian cells.^15^ However, with the modified surface properties and various colloidal stabilities in CCM, contradictory results of cytotoxicity have been reported.^13^ The biological application of CeNPs either as potential diagnostic or therapeutic agents depends on their preserved physicochemical properties after interacting with the cell culture media.^47–49^ CeNPs coated with polymers such as polyethylene glycol has been reported to exhibit more cytotoxicity than non-coated CeNPs towards breast cancer cells.^49^ Research reported *in vivo* study of erbium-doped CeNPs in treating acute liver injury by reducing the ROS products in the blood,^47^ but the effect of these CeNPs cytotoxicity after cellular uptake is unknown. Depending on the application, aiming for either more or less cytotoxicity towards the cells needs to be investigated by monitoring the CeNPs physicochemical properties after entering the cellular environment.

**Fig. 6:**
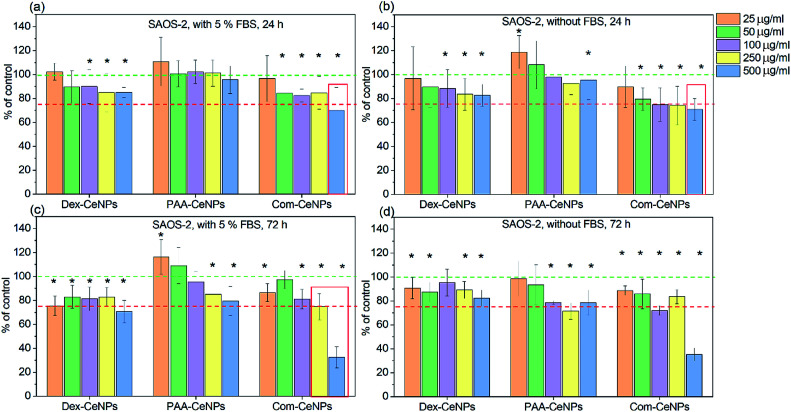
Cytotoxicity of CeNPs at different concentration when incubating with osteoblastic cell line SAOS-2: (a) CeNPs with cells for 24 hours in DMEM with 5% FBS supplement; (b) CeNPs with cells for 24 hours in DMEM without FBS; (c) CeNPs with cells for 72 hours in DMEM with 5% FBS supplement; and (d) CeNPs with cells for 72 hours in DMEM without FBS. Five different concentrations of CeNPs were tested in each set from left to right: 25, 50, 100, 250, and 500 μg ml^−1^. Green dashed lines indicate 100% control metabolic activity, while the red dashed lines indicate the level of toxicity for cells when their metabolic activity is decreased to 75%. The asterisk indicates data of significant difference with control (100%) at *p* < 0.05. The red frame indicates false-positive results (a strong interaction of NPs with the detection system).

We further determined the cytotoxicity of CeNPs using the human osteoblastic cell line (SAOS-2). The impact on the metabolic activity of osteoblasts was compared among CeNPs samples in Fig. 6, characterized by MTS assay (3-(4,5-dimethylthiazol-2-yl)-5-(3-carboxymethoxyphenyl)-2-(4-sulfophenyl)-2*H*-tetrazolium). It was found that within 24 hour incubation of osteoblast cells SAOS-2 with various concentrations of CeNPs under standard conditions (DMEM supplemented with 5% FBS), the Com-CeNPs caused a more significant decrease in metabolic activity of cells than Dex-CeNPs and PAA-CeNPs. This effect (dose-dependent) was even accentuated under non-standard conditions when FBS was absent. This decrease in cell viability lasted up to 72 hours when CeNPs at their highest concentration became cytotoxic (cell metabolic activity is lower than 75% of control). However, at high concentrations, Com-CeNPs strongly reacted with MTS reagent and can cause false positivity. We further checked the number of cells, which confirmed a dramatic cell decrease (cell death) (Fig. S1†). When cells were incubated with Dex-CeNPs for 24 hours, their metabolic activity was significantly decreased in comparison to control, but not to the cytotoxic level regardless of protein supplement. While in time, Dex-CeNPs decreased the metabolic activity of the cells even more, and in the presence of proteins, they reached the cytotoxic level. Surprisingly, their effect was not dosage-dependent. We have observed agglomeration for Dex-CeNPs in the presence of DMEM media with FBS within 72 hours (data not shown), indicating shorter shelf life and instability of Dex-CeNPs in solutions, which also corresponds to the value of their *Z*_P_ (absolute value of *Z*_P_ less than 20 mV indicates an unstable colloidal solution). As for PAA-CeNPs, incubation with osteoblasts for 24 hours was not harmful to the cells, but to the opposite, lower CeNPs concentration relatively increased cell metabolic activities regardless of the presence of FBS. For longer-term incubation (up to 72 hours), the addition of FBS helped to stabilize PAA-CeNPs and thus increased cell metabolic activity. The metabolic activity of osteoblasts in the presence of PAA-CeNPs and FBS was dose-dependent. At low NPs concentration (25 μg ml^−1^), it helped to increase the cell metabolic activity for up to 20%. This significant difference in cell viability suggested that testing of nanomaterials should be following their appropriate dosage and time of incubation.

**Fig. 7:**
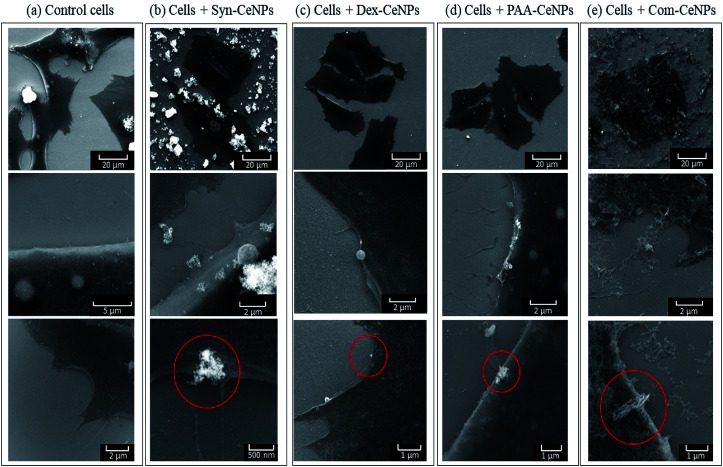
SEM images of SAOS-2 cells incubated (a) as control; and with (b) Syn-CeNPs; (c) Dex-CeNPs; (d) PAA-CeNPs; (e) Com-CeNPs. Presented images were all measured with electron beam energy at 15 keV. Red circles indicate the presence of CeNPs.

The interactions of all types of CeNPs with cells were further studied with SEM to observe the state of cell membranes and CeNPs agglomerates formation (Fig. 7). Compared with the controlled cells, the images of cells incubated with Syn-CeNPs and Com-CeNPs showed attached blanket-like aggregates onto the cell membranes even after extensive washing to remove unbounded CeNPs. These CeNPs were likely to be trapped and tangled with cell media and extracellular proteins. Cerium has a higher electron density than those elements from cells (carbon, nitrogen, or sulphur), thus showing as brighter parts in SEM images (red circles in Fig. 6). With the help of EDX, we further confirmed that the brighter parts contain Ce (Fig. S2†). In the case of Dex-CeNPs and PAA-CeNPs, much smaller particles were attached to the cell membrane, compared with Syn-CeNPs and Com-CeNPs. This observation further confirmed that polymer-coated CeNPs were stable in CCM, as described in the abovementioned sections. To this point, it is not clear yet how the cells uptake CeNPs depending on their surface coatings. It is well known that intracellular uptake and subsequent cell functions depend significantly on the size and surface properties of nanomaterials (surface potential or functional groups).^50^ Compared to the physicochemical properties of CeNPs, the colloidal behaviour of CeNPs after interaction with the cellular media environment determines the fate of CeNPs during cellular uptake. Further research is needed to clarify these factors influencing the internalization of CeNPs.

## Conclusions

In this study, we investigated the intrinsic and extrinsic factors of CeNPs surface properties that affect CeNPs colloidal stability, catalytic activities as enzyme mimetics, and their cytotoxicity in the CCM. We summarized the most critical factors influencing CeNPs colloidal stability in the CCM: CeNPs surface coating agents (neutral or charged polymers), solution properties (ionic strength), and the presence of macromolecules (amino acid and proteins). The first aspect determined the colloidal stabilization mechanism of CeNPs, while the latter two factors strongly influenced the behaviour of CeNPs in the CCM. Protein adsorption onto CeNPs was affected by the electrostatic forces generated either through CeNPs itself or the functional groups on the surfaces of CeNPs. Electrostatically stabilized CeNPs were more prone to agglomerate in the presence of high ionic strength of biological fluid, which further caused cell toxicity. The presence of polymers as steric repulsion on CeNPs surface created a colloidal system more resistant to particle agglomeration, and they also improved CeNPs stability *in vitro*. PAA had a minimum influence on CeNPs catalytic activity, without blocking the electron charge transfer pathway for CeNPs to act as enzyme mimetics. Furthermore, PAA-CeNPs exhibited a beneficial effect of cell metabolic activity improvement, indicating the vast potential application of PAA-CeNPs for biomedical applications in the future. Understanding the surface influence on CeNPs interaction with the biological environment gives us a further indication of how to design CeNPs with better stability and catalytic activity for medical therapies.

## Conflicts of interest

There are no conflicts to declare.

## Supplementary Material

RA-010-D0RA08063B-s001
