# Supplementary material for: Colloidal stability and catalytic activity of cerium oxide nanoparticles in cell culture media
Source: RSC Adv. 2020 Oct 27;10(65):39373–84. doi: 10.1039/d0ra08063b (PMC9057433; doi:10.1039/d0ra08063b)
Supplement: RA-010-D0RA08063B-s001 [file RA-010-D0RA08063B-s001.pdf]

Electronic Supplementary Material (ESI) for RSC Advances.
This journal is © The Royal Society of Chemistry 2020

Supporting Information

Colloidal Stability and Catalytic Activity of Cerium Oxide Nanoparticles in Cell Culture Media

Xiaohui Ju,*[a] Anna Fučíková,[b] Břetislav Šmíd,[a] Jaroslava Nováková,[a] Iva Matolínová,[a] Vladimír Matolín,[a] Martin Janata,[a] Tereza Bělinová,[c] Marie Hubálek Kalbáčová[c, d]

[a] Department of Surface and Plasma Science, Faculty of Mathematics and Physics, Charles University, V Holešovičkách 2, 180 00, Prague, Czech Republic

[b] Department of Chemical Physics and Optics, Faculty of Mathematics and Physics, Charles University, Ke Karlovu 3, 121 16, Prague, Czech Republic

[c] Biomedical Center, Medical Faculty in Pilsen, Charles University, Alej Svobody 1655/76, 323 00, Pilsen, Czech Republic

[d] Institute of Pathological Physiology, 1st Faculty of Medicine, Charles University, U Nemocnice 5, 128 53, Prague, Czech Republic

* Corresponding author email: xiaohui.ju@mff.cuni.cz.

Table S1. Composition of Dulbecco's Modified Eagle Medium (DMEM) used for mammalian cell in vitro experiments (Sigma Aldrich).

Components	Concentration (mg/L)
Amino acids	
Glycine	30
L-Arginine	84
L-Cystine 2HCl	63
L-Histidine hydrochloride-H_2O	42
L-Isoleucine	105
L-Leucine	105
L-Lysine	146
L-Methionine	30
L-Phenylalanine	66
L-Serine	42
L-Threonine	95
L-Tryptophan	16
L-Tyrosine	72
L-Valine	94
Vitamins	
Choline chloride	4
D-Calcium pantothenate	4
Folic Acid	4
Niacinamide	4
Pyridoxal hydrochloride	4
Riboflavin (B2)	0.4
Thiamine hydrochloride (B1)	4
i-Inositol	7.2
Inorganic salt	
Calcium Chloride ($CaCl_2$□$2H_2O$)	264
Magnesium Sulfate ($MgSO_4$) (anhyd.)	200
Potassium Chloride (KCl)	400
Sodium Bicarbonate ($NaHCO_3$)	3700
Sodium Chloride (NaCl)	6400
Sodium Phosphate monobasic (NaH_2PO_4□$2H_2O$)	141
Ferric Nitrate ($Fe(NO_3)_3$□$9H_2O$)	0.1
Other components	
D-Glucose (Dextrose)	1000
Sodium Pyruvate	110

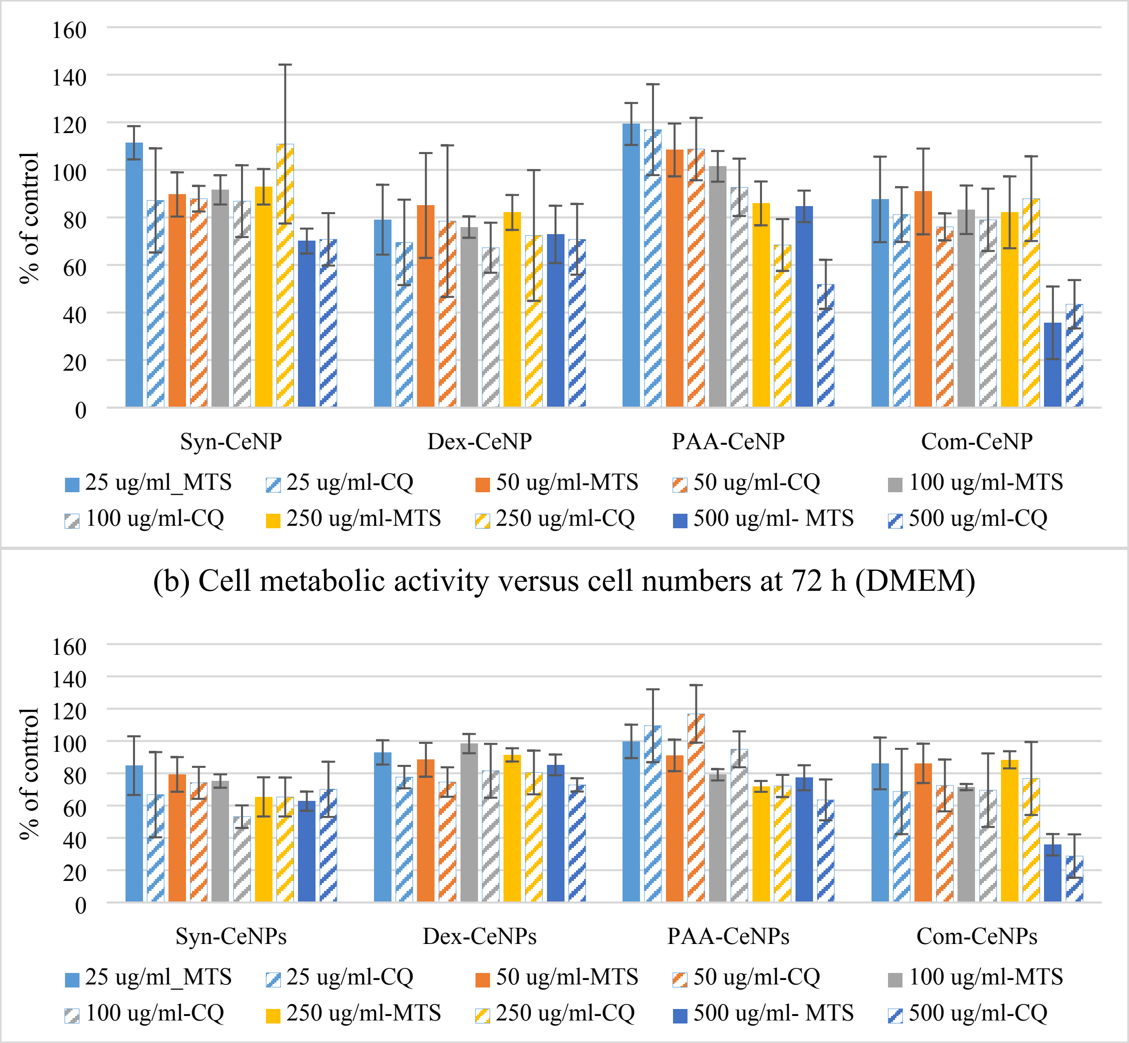

Fig. S1 Osteoblastic cells (SAOS-2) metabolic activity vs. cell number after 72 hours' incubation with various CeNPs concentrations in DMEM (a) with or (b) without 5 % FBS supplementation. Cell metabolic activity data measured by MTS assay (colorimetric-based method for quantification of cellular dehydrogenases activity) are labelled as MTS (solid colour), data of cell number measured by CyQuant assay (fluorescence-based method for quantification of cellular DNA content) are labelled as CQ (hatched colour). CyQuant Cell Proliferation Assays is a colorimetric fluorescence assay used to quantify cells. The DNA-CyQuant NF Cell Proliferation Assay Kit (Invitrogen) was used according to the manufacturer's manual. Fluorescence intensity was measured at an excitation wavelength of 485 nm and emission wavelength at 530 nm (TECAN Spark).

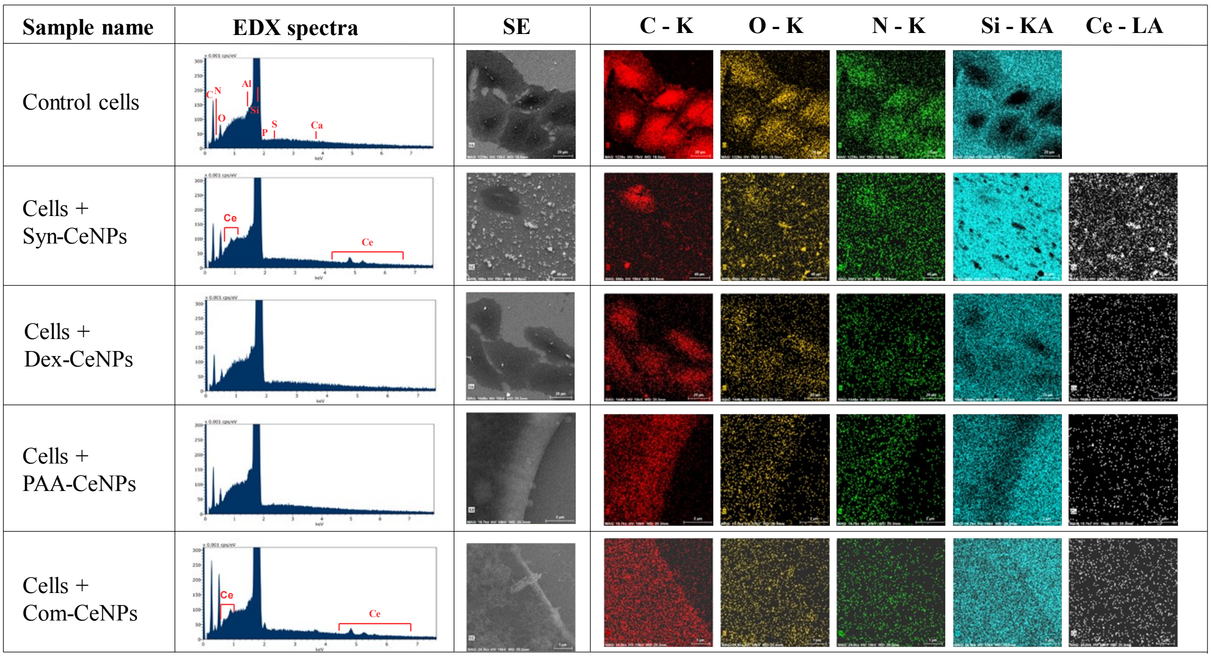

Fig. S2 EDX spectra of the osteoblast cell line (SAOS-2) incubated with various CeNPs. Cells without CeNPs were used as a control. EDX spectra were collected with a view field of 1 mm × 1 mm. The Secondary Electron images (SE) were presented as reference. Colours of red (C-K series), yellow (O-K series), green (N-K series), cyan (Si-KA) series and white (Ce-LA series) were represented in the figure, respectively. Ce signals were not able to be detected by EDX for Dex-CeNPs and PAA-CeNPs because the concentration of the nanoparticles was below the EDX detection limit.
